# The wasted chewing gum bacteriome

**DOI:** 10.1038/s41598-020-73913-4

**Published:** 2020-10-08

**Authors:** Leila Satari, Alba Guillén, Àngela Vidal-Verdú, Manuel Porcar

**Affiliations:** 1 Institute for Integrative Systems Biology I2SysBio, Universitat de València-CSIC, Catedrático José Beltrán 2, 46980 Paterna, Spain; 2 Darwin Bioprospecting Excellence SL, Catedrático Agustín Escardino 9, 46980 Paterna, Spain

**Keywords:** Environmental microbiology, Applied microbiology

## Abstract

Here we show the bacteriome of wasted chewing gums from five different countries and the microbial successions on wasted gums during three months of outdoors exposure. In addition, a collection of bacterial strains from wasted gums was set, and the biodegradation capability of different gum ingredients by the isolates was tested. Our results reveal that the oral microbiota present in gums after being chewed, characterised by the presence of species such as *Streptococcus* spp. or *Corynebacterium* spp., evolves in a few weeks to an environmental bacteriome characterised by the presence of *Acinetobacter* spp., *Sphingomonas* spp. and *Pseudomonas* spp. Wasted chewing gums collected worldwide contain a typical sub-aerial biofilm bacteriome, characterised by species such as *Sphingomonas* spp., *Kocuria* spp., *Deinococcus* spp. and *Blastococcus* spp. Our findings have implications for a wide range of disciplines, including forensics, contagious disease control, or bioremediation of wasted chewing gum residues.

## Introduction

Chewing gums may have been used for thousands of years, since wood tar from the Mesolithic and Neolithic periods have been found with tooth impressions, which suggests a role in teeth cleaning as well as its usage as early adhesives^1, 2^. The first modern chewing gum was introduced in the market in the late 19th^3^ and chewing gums are today vastly consumed worldwide: it is estimated that Iran and Saudi Arabia are the countries with the highest chewing gum consumption, where 80% of the population are regular chewing gum consumers^4^. Moreover, global online surveys on gum intake conducted in Europe^5^ and United States^6^ displayed similar chewing gum patterns among them, where more than 60% of adolescents and adults had chewed gums in the last 6 months before the survey and the mean intakes ranged from 1 to 4 pieces of chewing gum per day. Significantly, Hearty et al. (2014)^5^ reported the lowest chewing gum intake (46%) in the United Kingdom. Finally, the value of chewing gum trade has been estimated as more than 30 billion U.S. dollars in 2019^7^.

Chewing gums are generally composed of two phases: the water-insoluble phase (gum base) and the water-soluble phase, which can be made of sugar (sugar chewing gums) or sugar alcohols such as polyols (sugar-free chewing gums). Some chewing gums present a solid coat, which is involved in flavour release as well as in chewing gum protection to physicochemical agents, that can be specified as a third phase^8^. The main component of any chewing gum is the gum base (20–30%), that is not edible, nor digestible, but allows chewing, during which added flavours and sweeteners are released. Indeed, chewing gum can be chewed for a long period without any structural modifications because of the water-insoluble property of the gum base^9^. Gum base can be produced from either natural polymers, such as latex or waxes, or synthetic polymers, particularly polyvinyl acetate (15–45%)—a key ingredient in chewing gum formulation—and synthetic elastomers (10–30%) including co-polymers of butadiene-styrene, isobutylene-isoprene as well as, polyethylene, polyisobutylene and polyisoprene^8^. Hence, this inert part of the formula constitutes the support for the water-soluble components which consist of: (i) sweeteners, whether sugar or sugar alcohols that constitute the 60% of the chewing gum; (ii) humectants, such as glycerine; (iii) antioxidants, supplemented to avoid oxidation of other ingredients; (iv) colours, flavours and organic acids, added to define an specific taste of the chewing gum; and (v) optionally, “active” ingredients such as nicotine in chewing gums as an alternative to smoking^8, 9^. The particular amounts of these components in the chewing gum formula are a well-kept secret of each confectionery industry. As hinted before, sweeteners comprise more than half of the chewing gum composition. Sucrose, dextrose and glucose syrup are the most frequently used in sugar-containing gums. However, most of chewing gums present in the European market are sweetened with polyols (sugar alcohols) such as xylitol, sorbitol, mannitol, maltitol and isomalt^10, 11^ as well as artificial sweeteners such as aspartame^12^, all of which being labelled as sugar-free chewing gums. The effect of the sugar-free chewing gums in the control of dental disease, salivary pH, and the oral microbiome has been reported^10, 13–15^.

Wasted chewing gums are often improperly discarded and end up as long-lasting residues on both indoor and outdoor pavements and surfaces. Local councils spend millions of euros cleaning up gum residues from the pavement. For instance, it is estimated that in the UK the annual cost of cleaning up wasted gums form streets is almost 70 million euros^16^. Besides, there is a big concern regarding chewing gum residues stuck to historic buildings or art works, which contribute negatively to its conservation^17^. Moreover, the popularity of chewing gums as well as the widespread presence of those long-lasting residues have allowed using wasted chewed gums for human genetic analysis in criminology^18^ and archaeology^19, 20^. It has to be stressed, though, that beyond its contents in the consumer’s DNA, used chewed gum can contain an important fraction of the oral microbiome^21^, toxins^22^, and some opportunistic pathogens such as *Streptococcus* spp. and *Actinomyces* spp.^23, 24^*,* which remain trapped in the sticky residue, and whose survival over time has received only limited attention to date^17, 21^. Wasted chewing gums are considered as environmental pollutants, mainly for aesthetic reasons, and their removal from pavements can be economically expensive, and time-consuming^25^. To date, most studies aiming at improving wasted chewing gums cleaning have mainly focused on the production of less adhesive, water-soluble and degradable chewing gums^22, 26^.

The present work describes a complete characterization on the bacterial contents of wasted chewed gum, by using culture-dependent and -independent techniques. We have studied the microbial content of wasted chewing gums sampled in different locations worldwide as well as the distribution of bacteria depending on the depth (surface, intermediate and bottom layers of the residue) and conducted a dynamic study to shed light on the microbial succession that takes place in the chewing gum during the first weeks after its disposal on an outdoor surface. On the other hand, we have screened the biodegradation capability of the gum ingredients of a collection of bacterial strains we isolated from chewing gum residues. Our results have implications in fields such as criminology, contagious disease control, waste management and bioremediation.

## Results

### Bacterial communities in wasted chewing gums analysed through NGS

**Figure 1:**
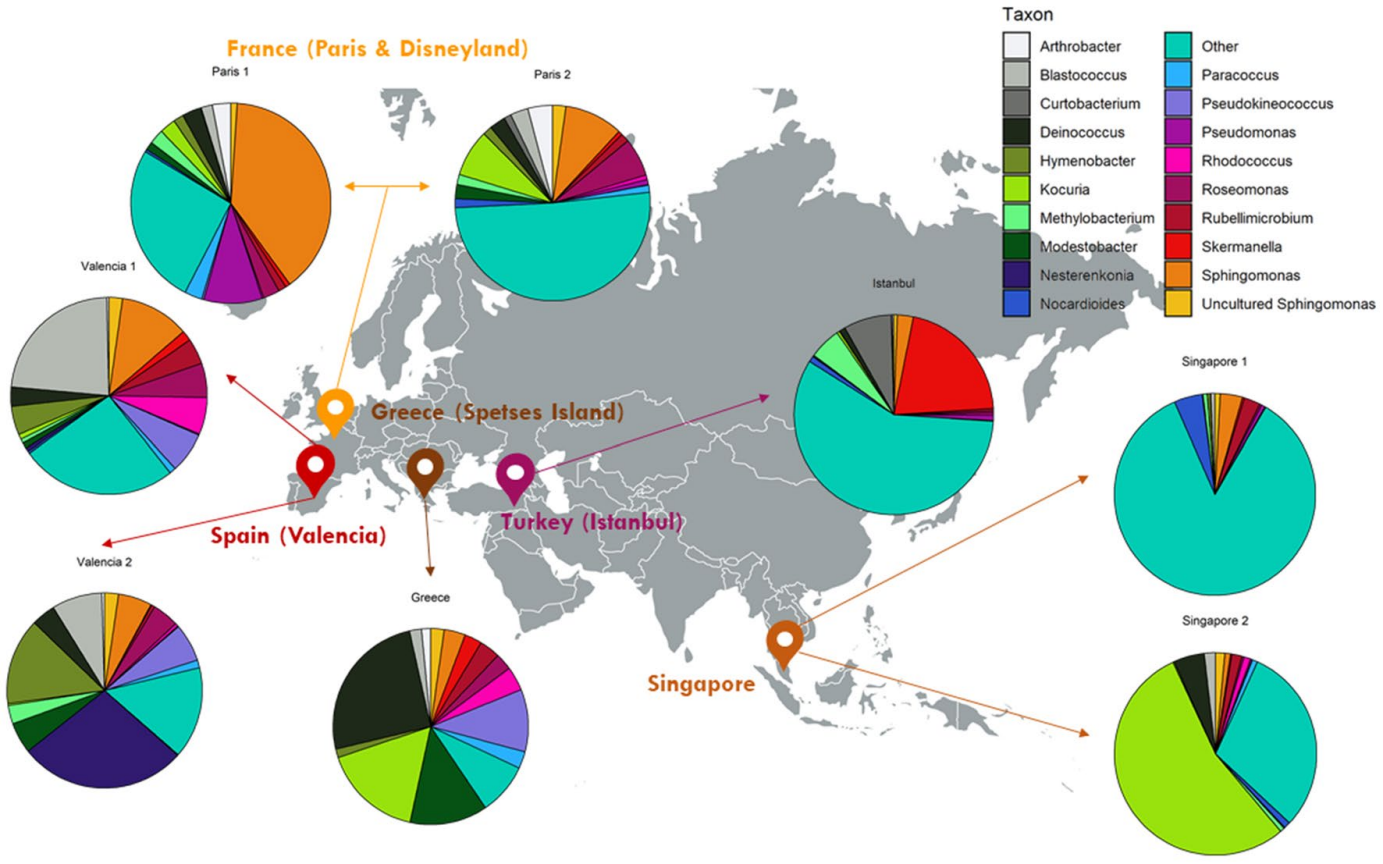
The taxonomic profiles of wasted chewing gum samples collected from outdoor locations in five countries. The most abundant taxa from each location are shown in pie charts. Figure created with *Adobe Illustrator CS6* (www.adobe.com).

The bacterial diversity of eight chewing gum samples collected in five different countries was analysed through NGS as described in the Materials and Methods section and the taxonomic profiles are shown in Fig. 1. Two chewing gum samples collected in Spain, France, and Singapore were analysed; while from Greece and Turkey one chewing gum sample was analysed. The bacterial profile deduced by the analysis of the pool of 16S rRNA genes reveals relatively similar bacterial profiles, but yet with differences in some genera. Interestingly, one of the samples from Singapore displayed a very high biodiversity with a total of 427 identified taxa, which results in a low relative abundance of the most common genera, being approximately the 15% in this sample. However, the number of samples per country being too small to draw conclusions at the geographic level, these results have to be considered as a first approach on the chewing gum bacteriome. Chewing gum samples from France (Paris) and Turkey (Istanbul) proved also rather diverse representing the most common genera the 49% and 42% of total abundance, respectively.

Although the eight samples exhibited an important variation among locations, several genera were found in all the analysed samples (*Kocuria*, *Sphingomonas*, *Deinococcus*, *Blastococcus*, *Skermanella*, *Hymenobacter*, *Modestobacter*, *Roseomonas*, *Rubellimicrobium*, *Methylobacterium*, Uncultured *Sphingomonas*, *Paracoccus* and *Nocardiodes*). Their relative abundance changed significantly among samples. The genus *Kocuria* with a 54.18% frequency and *Sphingomonas* with a 38.9%, were the most abundant genera in one of the samples from Singapore and France, whereas in other samples, such as Greece, the most frequent genera were *Deinococcus* with a 25.2% frequency, followed by *Kocuria*, *Pseudokineococcus* and *Modestobacter* with relative abundances from 10 to 16%. In samples from Valencia, other frequent genera were *Blastococcus*, *Nesterenkonia* and *Hymenobacter* with a 23.2%, 27.8% and 14.4% relative abundance, respectively. Finally, the genus *Skermanella* was the most abundant one in the chewing gum from Turkey (20.9%). *Arthrobacter* spp*.* was identified in all samples except the two samples from Singapore. Similarly, *Pseudomonas* spp*.* was found in all samples except in the one from Greece. Interestingly, in the two Mediterranean locations, three genera constituted approximately 25% of the biodiversity. These genera were: *Blastococcus* and *Nesterenkonia* from Valencia, Spain; and *Deinococcus* from Spetses Island, Greece*.* The presence of genus *Nesterenkonia* was found exclusively in the two samples from Spain. In addition, the genus *Curtobacterium* was found mainly in Turkey and in a lower abundance in France (Paris).

**Figure 2:**
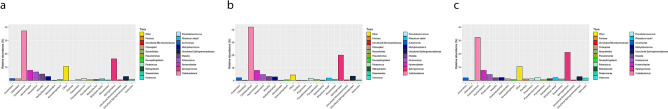
Comparison between the bacterial communities of three layers from a chewed gum residue. (**a**) Upper layer, (**b**) medium layer, and (**c**) bottom layer (described in M&M) showing small variation in biodiversity.

In one case, a single gum sample was collected and divided in situ in three different fractions that were processed independently. The sample, located in one of the outdoor car parks of the Scientific Park of the University of Valencia, was collected in three consecutive sections, corresponding to the upper, medium and bottom layers of the residue. As shown in Fig. 2, the taxonomic profiles of the three sub-samples proved almost identical in composition. The surface layer, which is the most exposed to environmental conditions, presented a higher abundance of chloroplasts. The main genus identified in the three samples was *Curtobacterium* with a relative abundance between 32–47%, being more frequent in the intermediate layer. Other, less frequent genera were *Sphingomonas*, which was the second most abundant genus and represented more than 16% of the biodiversity; *Hymenobacter* with an 8%; and *Kineococcus,* with a 4–6%. Finally, *Massilia*, an uncultured *Sphingomonadaceae*, *Methylobacterium*, *Aureimonas* and members of the *Rhizobium* clade (*Allorhizobium, Neorhizobium, Pararhizobium* and *Rhizobium*) had similar frequencies, of 1.5–5%. The remaining genera presented similar abundances and constituted approximately 9% of the total biodiversity in each layer.

### Colonisation process of wasted chewing gums

**Figure 3:**
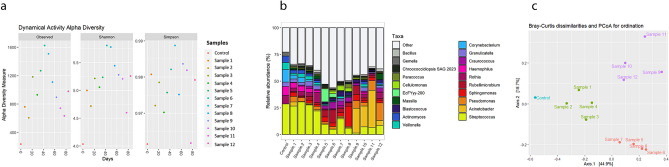
Dynamic experiment showing the variation of the chewed gum microbial communities in a twelve weeks period. (**a**) Alpha diversity shows the richness of the samples based on the Richness, Shannon, and Simpson index, (**b**) Clustered-Bar chart represents the modification of the oral bacterial profile over time, according to 16S rRNA gene monitoring. (**c**) Beta diversity (PCoA) based on Bray–Curtis illustrates correlations among the bacterial genera in different samples. Distances to the linear statistical correlation and the colours indicate the similarity of the microbial communities affected by environmental factors or removing time during the three-month period of the experiment.

A specific experiment was carried out to shed light on the microbial successions taking place in wasted chewing gum once discarded. Thirteen gums were chewed and placed on an outdoor pavement over a period of up to 12 weeks and high-throughput 16S rRNA sequencing was carried out to follow the dynamic changes in their bacterial contents. The shape of rarefaction curves at OTU level revealed a good coverage of the actual bacterial diversity (Supplementary Fig. 1). The high throughput sequencing and analysis of the 16S rRNA gene amplicons from all the samples revealed shifts in bacterial diversity in time (Fig. 3a), reaching the highest alpha diversity values after 6–8 weeks. The most abundant genera (Fig. 3b) included the genus *Streptococcus,* with a relative abundance of more than 25% in the control sample (which had its total DNA immediately extracted after being chewed). The relative frequency of this genus slowly decreased in time and reached its lowest abundance by the ninth week. Other frequent genera found in the control sample were the oral microbiome members *Rothia*, *Haemophilus*, *Corynebacterium*, *Veillonella*, *Actinomyces* and, to a lesser extent, *Granulicatella* and *Gemella*. All these genera remained detectable in the analysed samples throughout the whole experiment, but they clearly decreased in time, although with different temporal patterns. Interestingly, the relative abundance of non-oral, environmental genera, such as *Rubellimicrobium, Sphingomonas*, *Acinetobacter*, and *Pseudomonas* increased with the time the gums were kept outdoors, reaching maximum values of 12%, 19%, 23% and 16% respectively, after sixth, eighth, eleventh, and twelfth weeks each.

A Principal Coordinate Analysis (PCoA) proved that the bacterial communities in wasted chewing gums during a period of 12 weeks clustered in three groups (Fig. 3c). These groups corresponded to samples collected after 1–4; 5–7; and 9–12 weeks, thus indicating a clear association between taxonomic profile similarities and time of outdoor exposition.

**Figure 4:**
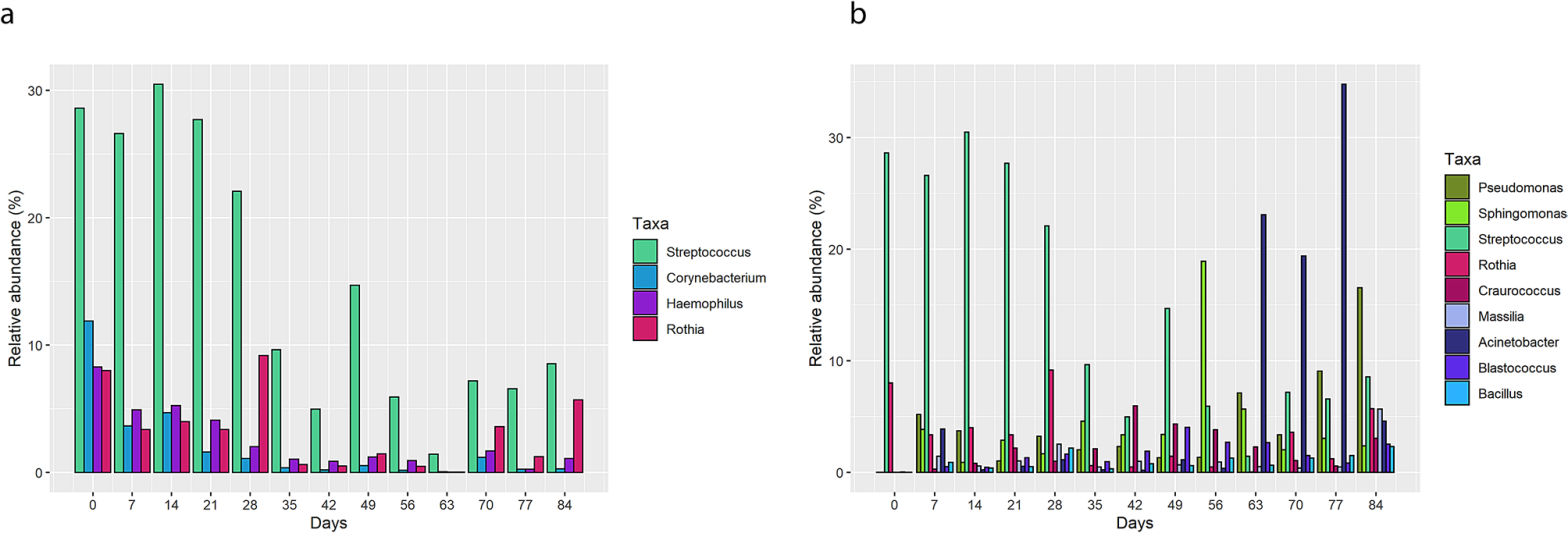
Comparison between the most frequent genera in (**a**) a control gum directly analysed after being chewed and (**b**) a sample taken after 12 weeks placed outdoors.

In order to identify the fate of the most common genera in the control sample in time, their relative abundance as well as that of the most frequent genera at the end (12th week) of the experiment were compared, as shown in Fig. 4. The four most abundant genera in the control sample were *Streptococcus*, *Corynebacterium*, *Haemophilus*, and *Rothia*, which constituted 56% of biodiversity of the sample (Fig. 4a). On the other hand, sample 12 included 9 main genera (51% of the reads) corresponding to environmental bacteria. In fact, and as Fig. 4 shows, the presence of the most abundant taxa in the control sample decreased significantly and was substituted by a soil-related profile in which the most frequent genera were *Acinetobacter*, *Sphingomonas* and *Pseudomonas*. The ecological succession of seven selected environmental genera (*Sphingomonas*, *Rubellimicrobium*, *Craurococcus*, *Granulicatella*, *Deinococcus*, *Hymenobacter*, and *Kocuria*) were monitored over time (Supplementary Fig. 2). All these genera, except *Granulicatella,* with a 4% abundance at the start, were not present in the control sample or only in a very low quantity, as the case of *Sphingomonas* and *Rubellimicrobium,* which showed an upward trend over time, peaking in the intermediate stages and stabilizing towards the end of the monitoring.

### Culture-dependent experiments

#### Strain collection from wasted chewing gums

Different chewing gum samples from the vicinity of our laboratory (Valencia, Spain) were ground and spread on different microbiological media. A total of 21 bacterial colonies, were selected, isolated and identified by 16S rRNA gene sequencing. The isolates selected from aerobically-incubated Petri dishes belonged to the following genera; *Curtobacterium* (S_1-1_), *Pantoea* (S_1-2_), *Microbacterium* (S_1-3_ and S_1-6_), *Pseudomonas* (S_1-4_), *Paenibacillus* (S_1-5_), *Arthrobacter* (S_2-1_, S_2-3_, S_2-6_), *Serinicoccus* (S_2-2_, S_2-5_), *Sphingomonas* (S_2-4_), *Aureimonas* (S_2-7_), *Bacillus* (S_3-1_), *Agrococcus* (S_3-17_), *Williamisia* (S_3-18_) (Supplementary Table 1). Five additional bacterial isolates (Supplementary Table 2), identified as members of the genera *Arthrobacter*, *Cellulosimicrobium*, *Sphingomonas*, *Terribacillus*, *Bacillus* were isolated under microaerophilic conditions.

#### Characterization of the degradation of chewing gum components

**Figure 5:**
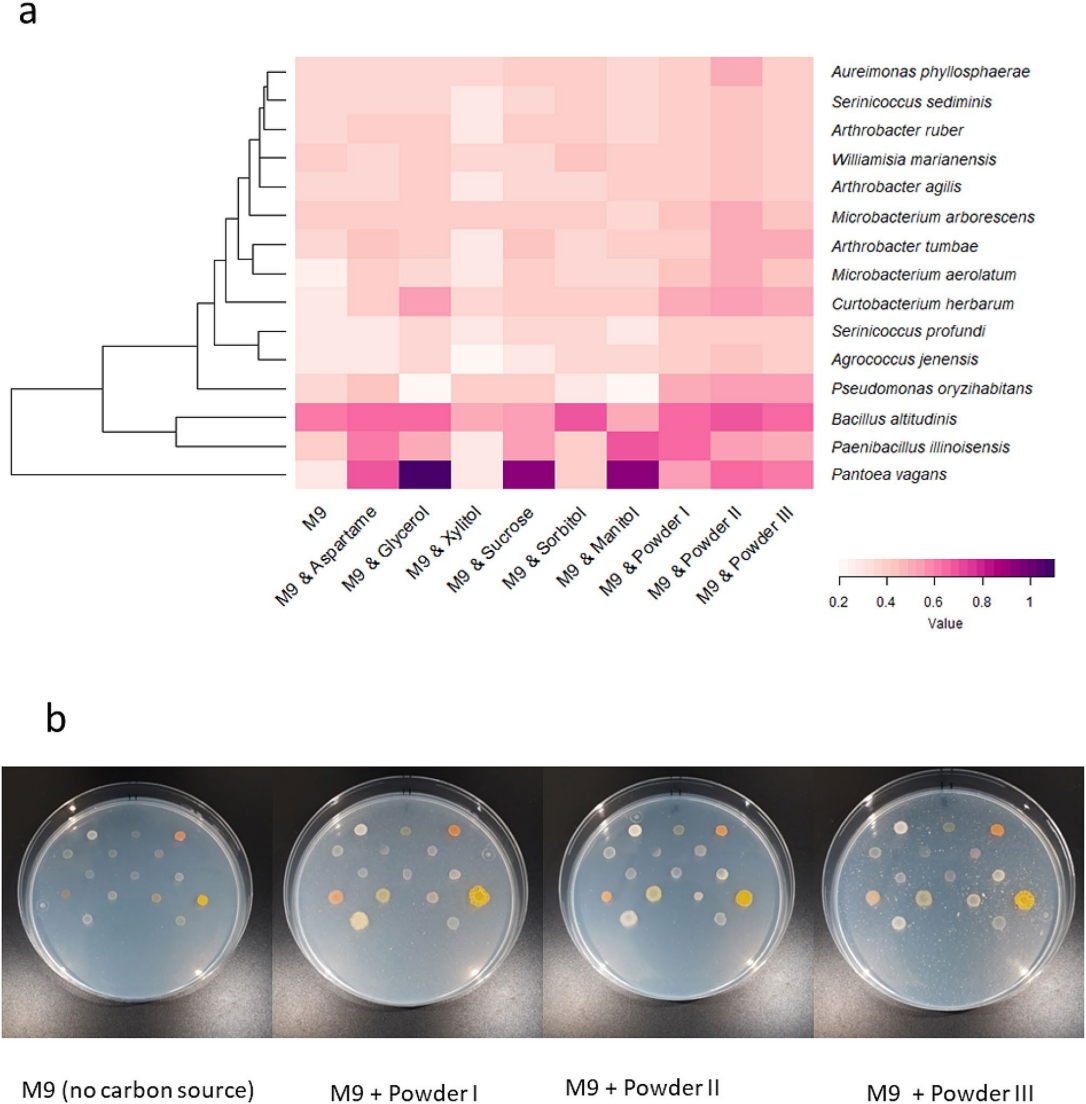
Degradation of various chewing gum ingredients by strains isolated from wasted chewing gums. (**a**) Heat map representing the ability to grow on different compounds of gum pieces as carbon sources. The phylogenetic tree and taxonomy of the chewed gum isolates based on 16S rRNA gene are shown in the left and right Y-axis, respectively. (**b**) The strains growth on modified M9 salt media enriched by chewing gum powder I, II, III, compared with the same medium without any carbon source as a control (left).

In order to identify possible degrading activities from the chewing gum isolates, a screening was carried out by growing those strains on minimal M9 medium supplemented with different possible components of chewing gum (Fig. 5a). In general, all the isolates grew better in the media supplemented with complete ground chewing gum. The *Bacillus altitudinis* strain was the only isolate that could grow well in minimal medium alone. *Pantoea vagans* showed the highest growth when sucrose, mannitol and glycerol were added to the medium as the only carbon source while it grew poorly in the presence of xylitol and sorbitol. *Paenibacillus illinoisensis* showed a wide spectrum of degradation of chewing gum components except xylitol and sorbitol. In fact, as shown in Fig. 5a, xylitol showed a significant inhibiting effect on the growth of the isolates. Finally, one strain, S_2–4_, with 98.54% similarity to *Sphingomonas insulae*, could not grow well on the solid media after isolation. The morphology of some strains was different depending on the type of chewing gum powder that was used as a carbon source for the selective media (Fig. 5b).

## Discussion

In this work, we describe the complete characterisation of the wasted chewing gum bacteriome. We report here that wasted chewing gums display a moderate diversity of bacterial population with variations among the samples analysed. Additionally, we show in a specifically designed assay that the oral community-based microbial pool is largely substituted, in a matter of few weeks, by an environmental bacteria-rich microbial community after a process of ecological successions.

The microbial community of the wasted gum samples consists of bacteria from phyla *Proteobacteria, Actinobacteria, Deinococcus-Thermus,* and *Bacteroides*. The most abundant families were *Sphingomonadaceae*, *Micrococcaceae*, *Geodermatophilaceae*, and *Deinococcaceae* and, at the genus level, *Sphingomonas*, *Kocuria*, *Blastococcus*, *Deinococcus*, and *Skermanella*; other frequent genera were *Nesterenkonia* and *Hymenobacter*. All those taxa have previously been reported as microbial communities associated with natural environments such as the phylloplane^27^, flowers^28^ or soil ecosystems^29^. The taxonomic profile we have detected is similar to abundant phyla on the surface of rocks in Maritime Antarctic glacier forefields^30^ and also the bacteriome of other outdoor surfaces such as photovoltaic, solar panels. In fact, solar panels are characterised by a desert-like bacteriome rich in *Sphingomonas*, *Deinococcus*, or *Hymenobacter*^31^. It is obvious that rocks or plant surfaces, solar panels and sun-exposed wasted chewing gums share similar ecological pressures in terms of irradiation, low water availability, thermal variations or oxidative stress.

*Streptococcus*, *Rothia*, *Haemophilus*, *Granulicatella*, *Corynebacterium*, *Veillonella*, *Actinomyces*, and *Gemella* were the most frequent genera in the control, chewed sample. These taxa are typical inhabitants of the mouth cavity, which bacteriome is composed of approximately 700 species from 185 genera^32, 33^. Our results reveal that the pool of oral microbiome bacteria present initially in the chewed samples is substituted after a colonisation process by environmental bacteria. However, some oral genera, specifically *Streptococcus*, was also detected at relatively high frequencies weeks after being used, as deduced by the colonisation experiment we conducted. Interestingly, *Streptococcus* was detected with very low relative abundance in most of the older-wasted chewing gum samples we studied, and not detected in samples of Greece and Singapore, suggesting that the stability in time of *Streptococcus* in the wasted chewing gum may not be more than a few weeks. Furthermore, other oral bacteria *Corynebacterium*, *Haemophilus*, *Veillonella* and *Gemella* were the most abundant genera in the control sample and these genera remained in the samples through the whole experiment, but their populations clearly decreased over time. The presence of *Corynebacterium* immediately decreased during the first month of outdoors incubation; however, it was observed with low intensity between 5–12 weeks. Furthermore, the lowest relative abundances from genera *Haemophilus*, *Veillonella* and *Gemella* were detected in sample 12. While, in comparison, the genus *Streptococcus* was the most frequent in this sample. These results suggest that wasted chewing gums constitute carriers of oral bacteria, some of which could be pathogens, even several weeks after being discarded. The biodiversity of the chewed gum microbial community increased after a few weeks outdoors, and the analysed samples after 6 or 9 weeks were more diverse and richer in genera such as *Craurococcus* and *Sphingomonas*, which were found in gum samples until the end of the period analysed. Other genera, such as *Cellulomonas* and *Rubellimicrobium* were observed in the gums of the same age as well, but they did not persist in time. Finally, the last samples analysed were rich in *Actinobacteria*, *Blastococcus* and other environmental bacteria. A possible explanation for this rearrangement may be that the transient taxa have degraded polymeric substrates remaining in the chewed gum to short carbon chains, and these simple carbon sources could then be hydrolysed by environmental bacteria. Additionally, the changes in abiotic factors such as pH, temperature, oxygen levels or water contents may play a role as selection forces driving community successions.

It is interesting to compare the bacterial profile of the chewing gums exposed outdoors for several weeks in the controlled experiment we performed with that of the “old” wasted chewing gums we sampled and analysed from different locations worldwide. *Pseudomonas*, *Sphingomonas*, *Rubellimicrobium*, and *Blastococcus* were present as the most abundant genera in almost all samples collected from different countries as well as the last sample of dynamic experiment (sample 12), which indicates that these taxa are fast, common colonisers of the wasted gums. Moreover, other environmental bacteria such as *Kocuria*, *Modestobacter*, *Deinococcus*, and *Roseomonas* were observed in all samples collected worldwide; however, they only constituted a small percentage of the microbial community in the dynamic samples labelled as “others” (data not shown). This strongly suggests that these taxa correspond to “second wave”, of slower but yet also cosmopolitan, microbial colonisers of the gum substrate.

Wasted gum being compact masses, the access of water and oxygen to the central part of the residues could be at least partially prevented, and it could thus be hypothesized that microbial communities would be different depending on their physical distance to the external environment. As a first approach to find out whether there was a variation in the bacterial composition across the depth of the waste, a single sample was divided into three successive layers that were analysed independently. Surprisingly, no significant differences were observed in the microbial communities of the different chewed gum layers of this sample. This result is striking, since even if the physico-chemical properties of a chewing gum may not change across its depth, UV radiation and water activity would be expected to be strong selective factors shaping a specific bacterial composition. *Sphingomonas* was detected in all three layers of the analysed chewed gum, as well as *Hymenobacter* and *Deinococcus,* all of which have been reported from extreme environments under strong desiccation and radiation conditions^34, 35^. Due to the limitation of the analysis of a single sample, further research including more samples is needed to address the colonisation of the interior of chewing gums.

Aspartame, mannitol, and glycerol were hydrolysed by several strains that we isolated from wasted chewing gums and identified as belonging to the species *Pantoea vagans*, *Paenibacillus illinoisensis*, and *Curtobacterium herbarum*. Aspartame was also remarkably degraded by *Pseudomonas oryzihabitans*, *Microbacterium arborescens*, and *Arthrobacter agilis*. The biodegradation of several artificial sweeteners including aspartame was recently studied^36, 37^, but the particular microbial keyplayers involved in the biodegradation process have been poorly studied. Degradation of mannitol and glycerol by *Pantoea vagans* and *Paenibacillus illinoisensis* has also previously been reported^38, 39^. Kuranishi et al.^40^ proved that *Pantoea* species can use mannitol as a substrate, by cultivating this genus on a designed semi-selective medium enriched with this compound. Another sweetener, sorbitol, was used as carbon source by one of our strains belonging to the genus *Aureimonas*. This genus has previously been found to hydrolyse a variety of carbon sources, such as carbohydrates, polyols and organic acids^41^. Our *Curtobacterium* strain was able to degrade almost all the tested chewing gum ingredients. Recently, Chase et al.^42^ reported that *Curtobacterium* spp. is able to degrade a wide range of carbohydrates, especially structural polysaccharides. It is thus tempting to hypothesize that bioaugmentation of some of the strains we characterized, specially *Curtobacterium herbarum,* may be used as a bioremediation strategy to contribute to remove chewing gum residues from contaminated pavements.

On the other hand, one of the main compositions of gum base are natural or artificial rubbers^43^. Previous research reported that these polymers could be degradable by bacteria; nevertheless, the biodegradation of rubbers is a long-term process^44^ since polymeric chains are cross linked by sulphur binding^45^. In this study, we have detected, either by culture-dependent or culture-independent techniques some taxa that have been previously described as rubber degraders. Tatangelo et al.^46^ showed that *Rhodococcus* can oxidize sulphur to sulphate, and desulphurization can facilitate the rubbers biodegradation. We also detected *Bacillus* spp. in both culture-dependent and -independent experiments, which has been described as a natural and synthetic rubber degrader^44, 47^. Interestingly, the population of *Bacillus* in the control sample of the dynamic experiment was very low; however, the presence of these bacteria gradually increased after several weeks of outdoors incubation. We also identified *Sphingomonas* spp. as one of the most frequent genera in culture-independent experiments and it was also isolated by culture-dependent methods, which could theoretically have a role in the biodegradation of polycyclic aromatic chains present in the gum base^43, 48^. Finally, *Corynebacterium* spp., have been reported as an opportunistic oral bacterium^49^ as well as a natural rubber degrader that needs direct contact with the surface of rubber particles^44^. In this research, this genus was detected as a component of the oral microbiome in the control and it remained during 12 weeks in a low-stable frequency that suggests it could play a role in long-term chewing gum biodegradation.

This study is the first report revealing from a holistic approach the bacterial composition of wasted chewing gum. Taken together, our results suggest that bacteria can play a role in the natural biodegradation of the chewing gum and may also be a source of strains with other biodegradation properties. The relative stability of the oral microbiome in a sun-irradiated outdoor space even after several weeks of outdoor and solar exposition raises concerns on the possible role of wasted chewing gums as long-term carriers of pathogenic microorganisms. On the other hand, the fact that the oral microbiome is partially maintained after many days suggest that, besides human DNA analysis, NGS aiming at determining the oral microbiome remaining in a chewing gum could hold potential for legal and forensic applications.

## Material and methods

### Chewing gum samples

Wasted chewing gums were collected directly from outdoor pavements in the vicinity of the Scientific Park of the University of Valencia as well from other locations worldwide. In particular, ten wasted gum samples were gathered from five countries (Fig. 1). All samples were removed from the pavement with a sterile scraper, and transported to the lab, where they were kept frozen at − 80 °C until required. In one case, a sample from Valencia (Spain), of approximately 3 mm thick, was sliced in situ in three different layers of roughly 1 mm each (upper, sun-irradiated, dark part; an intermediate part and the bottom fraction of the wasted gum, in contact with the pavement). The resulting three sub-samples were processed independently.

For the assays, two commercial sugar-free chewing gums were used, Orbit and Trident chewing gums. Both chewing gums were ground with a sterilized coffee grinder and they were added to a minimal medium as the sole carbon source. Gum powder I and II were prepared from the same source (Orbit gum pieces). The only difference between those two was that in the gum powder II, the white cortex layer was entirely removed before preparation in order to study whether bacteria only utilize the cortex layer, or they are able to degrade the whole compositions. Gum powder III was prepared from Trident brand.

### Media

M9 salt medium (ATCC 2511) was prepared (g l^−1^): Na_2_HPO_4_ 12.8, KH_2_PO_4_ 3.0, NaCl; 0.5, NH_4_Cl; 1.0, pH adjusted to 7.2. After autoclaving, the following filter-sterilized solutions were added to the medium; 2 ml of 1 M MgSO_4_ solution, 0.1 ml of 1 M CaCl_2_ solution, and 20 ml of a Glucose Solution (20% w/v). Glucose as a source of carbon was replaced, when necessary, by possible compositions of the chewing gum. M9 salt medium was modified and enriched with 2% (w/v) gum powder I as a selective medium. In addition to this medium, two rich media, TSA (composition in g l^−1^: Tryptone 15.0, Soya Peptone 5.0, NaCl 5.0, Agar 15.0) and LB (composition in g l^-1^: Tryptone 10.0, NaCl 10.0, Yeast Extract 5.0, Agar 15.0), were also used.

In order to study the biodegradation of the chewing gum compositions by isolates, the modified M9 salt medium (without glucose) was enriched with 2% (w/v) carbon sources including; Sucrose, Sorbitol, Mannitol, Xylitol, Aspartame, Gum powder I, II, III, and 2% (v/v) glycerol. Gum powders were used as other possible complementary ingredients of chewing gum.

### Culture-dependent approaches

Chewed gums were taken off with a sharp scraper from three locations in an open area from the Scientific Park of the University of Valencia, Spain. Two samples were incubated in liquid M9 salt solution (without glucose) supplemented with 2% (w/v) chewing Gum Powder I, at room temperature orbital shaking (150 rpm) for 24 and 48 h, respectively. The third sample was directly plated on the same solid medium after resuspension. Several serial dilutions were prepared and cultured on the selective media. Plates were incubated at room temperature for a week under aerophilic, anaerobic and microaerophilic conditions. Isolated colonies were selected based on their shape and colour and they were re-streaked on fresh media. Pure isolates were resuspended in 20% glycerol (v/v) and cryo-preserved at − 80 °C.

Polymerase chain reaction was performed with universal primers 8F (5′-AGAGTTTGATCCTGGCTCAG-3′) and 1492R (5′-CGGTTACCTTGTTACGACTT-3′) to amplify 16S rRNA gene. A loopful of bacterial cells was resuspended in 100 µl MilliQ water, pre-incubated at 100 °C for 10 min, and 1 µl of each bacterial suspension was used as DNA template. Thermal cycler program was set as the following procedure; Initial step of incubation at 95 °C for five min followed by PCR amplification (30 cycles of 30 s at 95 °C, 30 s at 54 °C, 30 s at 72 °C), and final step at 72 °C for 10 min. PCR products were monitored by 1% agarose gel electrophoresis to confirm the amplification of the 16S rRNA gene fragment amplicon. Next, dsDNA was purified from the PCR products and resuspended in 10 μl MilliQ water. 16S rRNA Sanger sequencing was performed by tagging with BigDye Terminator v3.1 Cycle Sequencing Kit (Applied Biosystems, Carlsbad, CA, USA), at the Sequencing Service (SCSIE) of the University of Valencia. All sequences were edited and compared with EzBioCloud online database (https://www.ezbiocloud.net/).

### Carbon source use

To study the ability of the bacterial strains isolated from wasted chewing gum to degrade different components, chewing gum sweeteners and glycerol were added as carbon sources to the minimal medium (described in media section). Moreover, three different media containing Gum Powder I, II, III separately were prepared to observe its biodegradation. For each isolated strain, a dilution of 0.2 optical density (OD_600_) was prepared with fresh liquid M9 medium. Then 2 µl of the dilutions were spread on the three different media aforementioned, in three replicates. The plates were incubated at room temperature under aerobic condition for five days. Also, the minimal M9 medium without any carbon source was used as the control medium.

### Ecological succession and bacterial colonisation

A study of the microbial colonisation of wasted chewing gum under controlled conditions was carried out. The study protocol was approved by the board of directors of The Institute for Integrative Systems Biology (I2SysBio) and was conducted under the guidelines of Helsinki declaration of 2013. Informed consent of the volunteer was obtained prior the study. A healthy volunteer (36 years old female) chewed two chewing gum pieces every day for 30 min. The first chewed gum was stored in − 80 °C as a control of the oral microbiome. Twelve chewed gums processed this way were placed on the sidewalk of the Scientific Park (University of Valencia, Spain) on an outdoor, sun-oriented location. The experiment was conducted in mid-June, and wasted gums were processed as described for 12 consecutive days. Then, they were picked up in intervals of one week during a total period of twelve weeks. Total DNA was extracted from each sample and 16S rRNA metagenomic analysis was carried out.

### 16S rRNA sequencing

The following procedures for DNA extraction and 16S rRNA sequencing were performed in all the experiments. All chewing gum residues were frozen after sampling and stored in − 80 °C at least for overnight. Frozen samples were ground to fine powder and added to 1 ml PBS buffer. Mixtures of wasted gum and PBS buffer were frozen at − 20 °C overnight. Then, 10 glass plating beads (3 mm diameter) were added to the tubes and mixed with a vortex for 3 min. Samples were let at room temperature for 2 min, and then 500 µl of the supernatant was transferred to a 2 ml micro-tube. DNA extraction was then carried out as described by Latorre et al. (1987)^50^, and the extracted DNA was quantified through Qubit dsDNA HS Assay kit (Qubit 2.0 Fluorometer, Q32866).

The conserved regions V3 and V4 (459 bp) of the 16S rRNA gene were then amplified using forward and reverse primers: 5′-TCG TCG GCA GCG TCA GAT GTG TAT AAG AGA CAG CCT ACG GGN GGC WGC AG 3′ and 5′- GTC TCG TGG GCT CGG AGA TGT GTA TAA GAG ACA GGA CTA CHV GGG TAT CTA ATC C -3′, respectively^51^. Amplification was carried out using the KAPA HiFi HotStart ReadyMix PCR kit (KK2602) and the following PCR cycle: initial denaturation at 95 °C for 3 min; 25 cycles of amplification (30 s at 95 °C, 30 s at 55 °C, 30 s at 72 °C); and 5 min of extension at 72 °C. Amplicons were mixed with Illumina sequencing adaptors and dual-index barcodes (Nextera XT index kit v2, FC-131-2001). Libraries were normalized and merged before the sequencing. Then, the pool containing indexed amplicons was loaded onto the MiSeq reagent cartridge v3 (MS-102-3003), spiked with 10% PhiX control to enhance the quality of the sequencing. Finally, paired-end sequencing (2 × 300 bp) was carried out on the Illumina MiSeq sequencing system. Illumina outcomes were analysed via Qiime2 software^52, 53^. The Demux plugin was used to assess the quality of reads, and the Qiime2-integrated Dada2 pipeline was employed for trimming and joining the sequences, removing chimeras and detecting sequence variants (> 99.9% of similarity). The classify-Sklearn module (feature-classifier plugin) was applied for assessing the taxonomy of each sequence variant, using the SILVA (v. 132) database as reference.

Data was subsequently analysed by different R packages. Rarefaction curves were constructed with the iNEXT and ggiNEXT functions (iNEXT)^54^. Alpha diversity plots were generated by employing the plot_richness function (Phyloseq)^55^ based on the richness, Shannon and Simpson diversity indexes. PCoA were created with the plot_ordination function (phyloseq) using Bray–Curtis dissimilarities as distance method. Finally, heatmap was constructed by the heatmap.2 function (gplots).

## Supplementary information

Supplementary file1

## Data Availability

Raw reads are available at NCBI’s Sequence Read Archive (SRA) (Bioproject Accession PRJNA641111).
