# Supplementary material for: The wasted chewing gum bacteriome
Source: Sci Rep. 2020 Oct 8;10:16846. doi: 10.1038/s41598-020-73913-4 (PMC7545173; doi:10.1038/s41598-020-73913-4)
Supplement: Supplementary file 1 — Supplementary file1 [file 41598_2020_73913_MOESM1_ESM.pdf]

Supplementary material

Leila Satari, Alba Guillén, Àngela Vidal-Verdú and Manuel Porcar. **“The wasted chewing gum bacteriome”**

Supplementary Figures

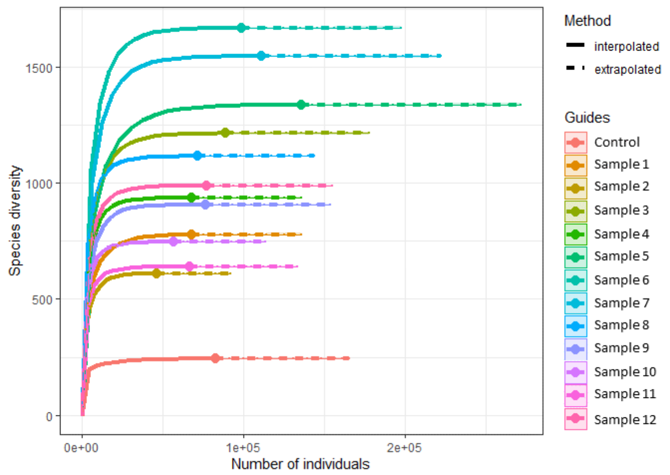

Supplementary Figure 1. Rarefaction curves at OTU level were plotted in order to assed the depth of the 16S sequencing process and observed the species diversity in each sample.

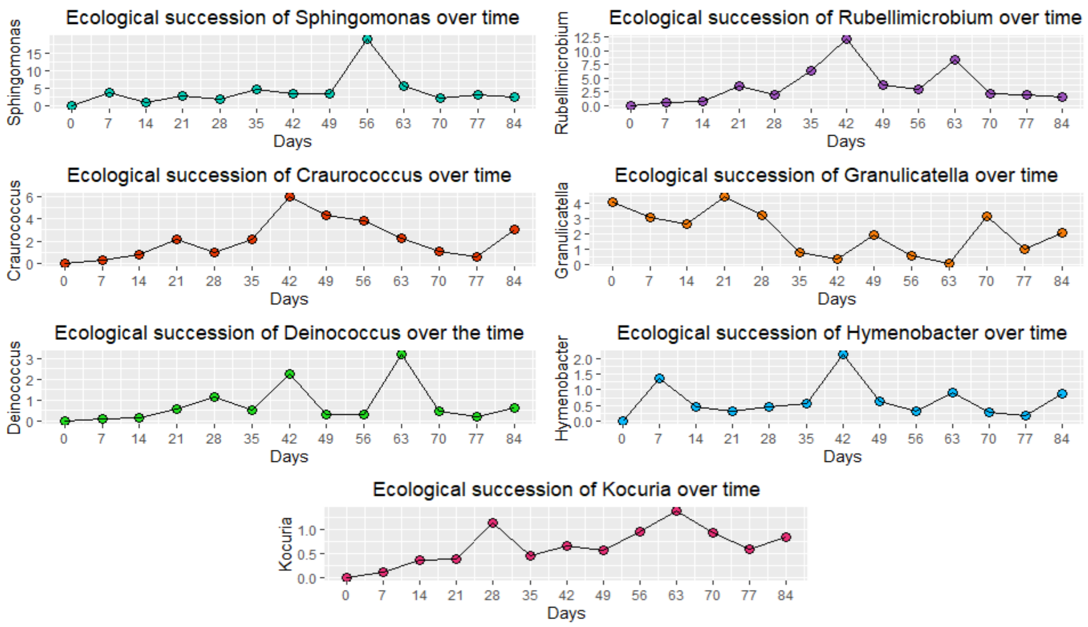

Supplementary Figure 2. Ecological succession of most abundant environmental genera in a dynamic study.

Supplementary Tables

Supplementary Table 1. Identification of wasted chewing gum aerophilic isolates.

ISOLATE	SIMILARITY (%)	IDENTIFICATION
**SAMPLE1- NO1**	98.45	*Curtobacterium herbarum*
**SAMPLE1- NO2**	98.60	*Pantoea vagans*
**SAMPLE1- NO3**	99.86	*Microbacterium arborescens*
**SAMPLE1- NO4**	99.62	*Pseudomonas oryzihabitans*
**SAMPLE1- NO5**	99.16	*Paenibacillus illinoisensis*
**SAMPLE1- NO6**	99.07	*Microbacterium aerolatum*
**SAMPLE2- NO1**	99.28	*Arthrobacter tumbae*
**SAMPLE2- NO2**	99.69	*Serinicoccus sediminis*
**SAMPLE2- NO3**	99.50	*Arthrobacter ruber*
**SAMPLE2- NO4**	98.77	*Sphingomonas insulae*
**SAMPLE2- NO5**	99.12	*Serinicoccus profundi*
**SAMPLE2- NO6**	99.58	*Arthrobacter agilis*
**SAMPLE2- NO7**	97.91	*Aureimonas phyllosphaerae*
**SAMPLE3- NO1**	99.75	*Bacillus altitudinis*
**SAMPLE3- NO17**	98.84	*Agrococcus jenensis*
**SAMPLE3- NO18**	99.31	*Williamisia marianensis*

Supplementary Table 2. Identification of wasted chewing gum microaerophilic isolates.

ISOLATE	SIMILARITY (%)	IDENTIFICATION
**MICROAEROPHILE- NO1**	99.51	*Arthrobacter ruber*
**MICROAEROPHILE- NO2**	99.18	*Cellulosimicrobium cellulans*
**MICROAEROPHILE- NO3**	98.90	*Sphingomonas insulae*
**MICROAEROPHILE- NO4**	99.59	*Terribacillus goriensis*
**MICROAEROPHILE- NO5**	99.54	*Bacillus simplex*
